# Glucose metabolism in tumor-associated macrophage plasticity and cancer immunity

**DOI:** 10.3389/fcell.2026.1885974

**Published:** 2026-07-20

**Authors:** Bang Liu, Jin Wang, Xiulin Jiang, Xiangyu Chen

**Affiliations:** 1 Hunan Provincial Key Laboratory of Regional Hereditary Birth Defects Prevention and Control, Changsha Hospital for Maternal and Child Care, Hunan Normal University, Changsha, Hunan, China; 2 College of Life Science, University of Chinese Academy of Sciences, Beijing, China

**Keywords:** glucose metabolism, glycolysis, HIF-1α, immunometabolism, lactate, metabolic reprogramming, pentose phosphate pathway (PPP), pyruvate metabolism

## Abstract

Tumor-associated macrophages (TAMs) are key immune cells in the tumor microenvironment and play critical roles in tumor progression, immune escape, and therapeutic response. Their functional plasticity is closely regulated by metabolic reprogramming, particularly glucose metabolism. Glucose-related pathways, including glycolysis, gluconeogenesis, the pentose phosphate pathway, glycogen metabolism, and pyruvate/lactate metabolism, influence TAM polarization, cytokine production, phagocytosis, antigen presentation, and T cell interactions. In many tumors, enhanced glycolysis and lactate accumulation promote M2-like TAM polarization and suppress CD8^+^ T cell activity, whereas certain metabolic programs may support M1-like anti-tumor functions under specific conditions. This mini review summarizes major glucose metabolic pathways involved in TAM regulation, highlights their context-dependent pro- and anti-tumor roles, and discusses therapeutic strategies for reprogramming TAM metabolism to improve anti-tumor immunity and immunotherapy response.

## 1 Introduction

Tumor-associated macrophages (TAMs) are among the most abundant immune cells in the tumor microenvironment (TME) and play essential roles in tumor progression, immune escape, angiogenesis, metastasis, and therapeutic response (Gao et al., 2022). Because macrophages are highly plastic, TAMs should not be described by a rigid M1/M2 classification (Wang et al., 2023). Instead, they exist along a functional spectrum. M1-like TAMs are generally associated with inflammatory cytokine production, antigen presentation, phagocytosis, and anti-tumor T cell responses, whereas M2-like TAMs often promote immune suppression, tissue remodeling, angiogenesis, and tumor growth (Gunassekaran et al., 2021). Therefore, understanding the mechanisms that regulate TAM plasticity is important for improving cancer immunotherapy.

Metabolic reprogramming is a major mechanism controlling TAM phenotype and function (Wang et al., 2022). Among different metabolic pathways, glucose metabolism is particularly important because it provides energy, biosynthetic intermediates, and immune-regulatory metabolites (Wang et al., 2022). Several glucose-related pathways contribute to TAM functional plasticity, including glycolysis, lactate and hypoxia-associated metabolic regulation (Wang Y. et al., 2024; Han et al., 2023; Wang W. et al., 2024). Glycolytic enzymes such as PKM2, ALDOA, PGK1, PGAM1, and ENO family proteins can regulate tumor–macrophage crosstalk, while lactate acts as an important immunoregulatory metabolite that suppresses CD8^+^ T cell activity and promotes tumor progression (Yu Y. et al., 2024; Yu S. et al., 2024; Yan et al., 2026; Islam Khan et al., 2022). Hypoxia further reshapes TAM glucose metabolism through HIF-1α-mediated regulation of glucose uptake, glycolysis, lactate production, and extracellular acidification (Pang et al., 2023). These metabolic changes interact with tumor-derived signals, stromal cells, inflammatory pathways, and therapy pressure to reinforce TAM heterogeneity in the TME. In this mini review, we summarize how major glucose metabolic pathways regulate TAM polarization, immune suppression, tumor progression, and therapeutic response.

## 2 Classification and functional characteristics of TAMs

**FIGURE 1:**
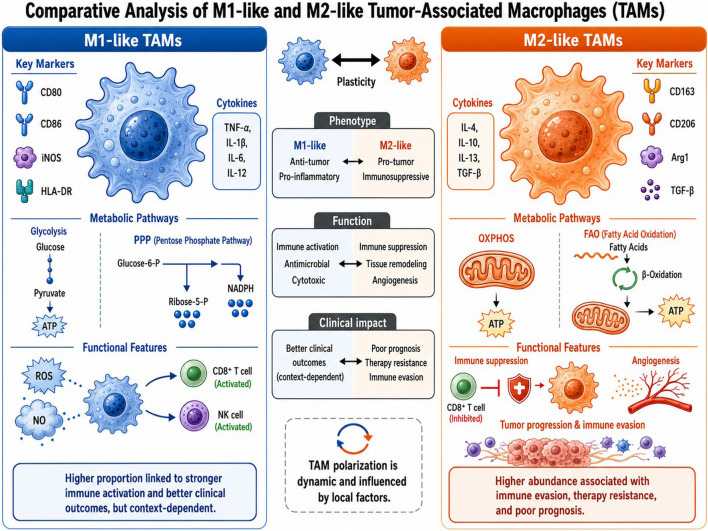
Comparative features of M1-like and M2-like tumor-associated macrophages. M1-like TAMs are characterized by markers such as CD80, CD86, iNOS, and HLA-DR, secretion of pro-inflammatory cytokines, and increased glycolysis and pentose phosphate pathway activity. Functionally, they promote ROS and NO production, activate CD8^+^ T cells and NK cells, and are generally associated with anti-tumor immunity. In contrast, M2-like TAMs express CD163, CD206, Arg1, and TGF-β, rely more on oxidative phosphorylation and fatty acid oxidation, and promote immune suppression, angiogenesis, tumor progression, and therapy resistance. TAM polarization is dynamic and shaped by local signals within the TME.

Although TAMs display a broad and continuous phenotypic spectrum, they are often discussed as M1-like pro-inflammatory, anti-tumor TAMs and M2-like immunosuppressive, tumor-promoting TAMs (Yan and Wan, 2021). To provide a clear framework for this review, we first summarize their major differences in markers, cytokine profiles, metabolic preferences, immune functions, and clinical implications **(** Figure 1 **)**.

### 2.1 M1-like TAMs

M1-like TAMs, also known as classically activated macrophages, are mainly induced by pro-inflammatory signals such as interferon-gamma (IFN-γ) and lipopolysaccharide (LPS) (He et al., 2025). They usually express high levels of cluster of differentiation 80 (CD80), CD86, and inducible nitric oxide synthase (iNOS), and HLA-DR, and produce pro-inflammatory cytokines, including TNF-α, IL-1β, IL-6, and IL-12. Functionally, M1-like TAMs support antigen presentation, generate reactive oxygen species (ROS) and nitric oxide (NO), and promote the activation of cytotoxic CD8^+^ T cells and natural killer (NK) cells. Therefore, they are generally considered to have anti-tumor immune activity (He et al., 2025).

### 2.2 M2-like TAMs

M2-like TAMs, also called alternatively activated macrophages, are commonly induced by interleukin-4 (IL-4), interleukin-10 (IL-10), interleukin-13 (IL-13), and transforming growth factor-beta (TGF-β) (Wang S. et al., 2024). They are characterized by increased expression of CD163 and CD206, Arg1, and TGF-β. In contrast to M1-like TAMs, M2-like TAMs usually suppress anti-tumor immunity and support tumor progression. They promote angiogenesis, extracellular matrix remodeling, tissue repair-like responses, and immune escape (Chen et al., 2025). Metabolically, M2-like TAMs preferentially use oxidative phosphorylation (OXPHOS) and fatty acid oxidation (FAO). Their immunosuppressive phenotype can also be strengthened by tumor-derived metabolites such as lactate and pyruvate (Chen et al., 2025). Functionally, M2-like TAMs recruit regulatory T cells (Tregs), inhibit CD8^+^ T cell activity, and form reciprocal pro-tumor loops with cancer cells. A high abundance of M2-like TAMs is often linked to immune evasion, therapy resistance, and poor prognosis (Chen et al., 2025).

## 3 Glucose metabolism in TAMs: functions and mechanisms

**FIGURE 2:**
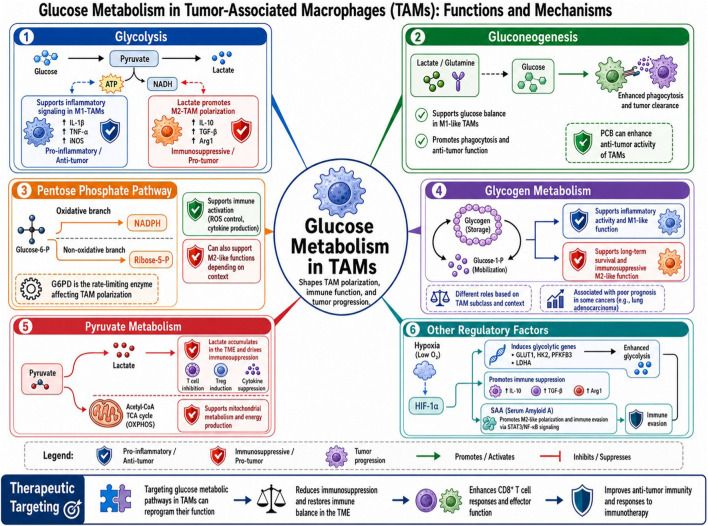
Glucose metabolic pathways regulating TAM polarization and immune function. Glucose metabolism controls TAM plasticity through multiple interconnected pathways. Glycolysis supports inflammatory activity in M1-like TAMs but can also promote M2-like polarization through lactate accumulation. Gluconeogenesis helps maintain glucose balance and phagocytic activity, whereas the pentose phosphate pathway provides NADPH and ribose-5-phosphate to support redox control, cytokine production, and context-dependent TAM functions. Glycogen and pyruvate metabolism further regulate TAM survival, mitochondrial activity, and lactate-mediated immunosuppression. Hypoxia, HIF-1α signaling, and inflammatory mediators such as SAA also reshape TAM metabolism and contribute to immune evasion. Targeting these metabolic pathways may reprogram TAMs, reduce immunosuppression, and enhance anti-tumor immunity and immunotherapy responses.

Building on this phenotypic comparison, we further illustrate how distinct glucose metabolic pathways shape TAM polarization and immune activity in the TME (Yan and Wan, 2021). These pathways, including glycolysis, gluconeogenesis, the pentose phosphate pathway, glycogen metabolism, pyruvate metabolism, and hypoxia-related regulatory signals, collectively link metabolic adaptation to macrophage plasticity, immune suppression, and therapeutic response **(** Figure 2 **)**.

### 3.1 Glycolysis

Glycolysis is a cytosolic pathway that converts glucose into pyruvate, producing ATP and NADH (Kierans and Taylor, 2024). In the hypoxic and nutrient-limited TME, TAMs often increase glycolytic activity (Kierans and Taylor, 2024). For example, glycolysis can support inflammatory signaling in M1-like TAMs, whereas tumor-associated glycolytic products, especially lactate, may promote M2-like polarization and immune suppression (Kierans and Taylor, 2024). Thus, glycolysis has a dual role in TAM biology, depending on the cellular source, tumor context, and downstream metabolites. Besides lactate, other glycolysis-linked metabolites may also exert context-dependent effects on TAM biology. For example, pyruvate can either be converted into lactate to support immunosuppressive signaling or enter mitochondrial metabolism to sustain macrophage activation (Yu Y. et al., 2024; Zhang D. et al., 2019). In hepatocellular carcinoma, TAM-derived circPETH-147aa promotes PKM2-mediated ALDOA-S36 phosphorylation, which enhances glycolysis in cancer cells and suppresses CD8^+^ T cell metabolism, thereby promoting metastasis and immune escape (Lan et al., 2025). Similarly, ALDOA-positive stem-like melanoma cells, often linked to BRAF/NRAS mutations, show enhanced glycolytic activity and altered immune interactions, indicating that glycolytic enzymes may connect tumor stemness with immune remodeling (Lan et al., 2025). In pro-tumor macrophages, increased glycolysis can support M2-like polarization by providing metabolic intermediates and promoting lactate accumulation within the TME. Lactate further reinforces immunosuppressive macrophage programs, reduces CD8^+^ T cell activity, and contributes to tumor growth, invasion, and resistance to immunotherapy (Yang et al., 2022; Xu et al., 2024). For instance, IL-6 secreted by M2-like TAMs activates PDPK1 in tumor cells and promotes PGK1 phosphorylation at T243, thereby increasing glycolytic flux and tumor growth (Zhang et al., 2018). In contrast, TRIM50 suppresses glycolysis in gastric cancer by ubiquitinating PGK1, reducing lactate production and reduced M2-like TAM polarization (Gu et al., 2024). Other glycolytic regulators also participate in TAM-tumor crosstalk. In pancreatic cancer, TAM-enriched lncRNA SNHG17 sponges miR-628-5p and promotes PGK1 T168 phosphorylation, which enhances tumor glycolysis and supports M2-like TAM polarization (Lin et al., 2023). In triple-negative breast cancer, PGAM1 promotes glycolysis and favors an immunosuppressive TME with increased M2-like TAMs and Tregs. Inhibition of PGAM1 remodels the immune microenvironment and improves anti-PD-1 therapy (Zhang et al., 2024). PGAM1 is a key glycolytic enzyme that catalyzes the conversion of 3-phosphoglycerate to 2-phosphoglycerate and is frequently upregulated in multiple tumor types, including triple-negative breast cancer (Zhang et al., 2024). Increased PGAM1 expression enhances glycolytic flux and supports tumor proliferation, migration, invasion, and poor prognosis. Importantly, PGAM1 also contributes to immune remodeling of the TME (Zhang et al., 2024). Inhibition of PGAM1 reduces tumor glycolysis and reshapes the immunosuppressive microenvironment by decreasing M2-like TAMs and Tregs, increasing anti-tumor immune activity, and enhancing the efficacy of anti-PD-1 therapy. These findings suggest that PGAM1 functions not only as a glycolytic enzyme but also as an immunometabolic regulator of tumor progression and therapeutic response (Zhang et al., 2024). Consistently, in colorectal cancer, PGAM1 promotes M2-like polarization and reduces CD8^+^ T cell infiltration, while the PGAM1 inhibitor HKB99 reprograms TAMs and enhances anti-PD-1 efficacy (Wang C. et al., 2024). These findings suggest that PGAM1 may be more than a glycolytic enzyme; it may also act as an immunometabolic regulator.

ENO family enzymes provide another example of how glycolysis-related molecules affect TAM function. In glioblastoma, tumor-derived ENO1 activates the TLR4/PI3K-Akt/ERK-SPHK1 pathway, promoting tumor invasion and inducing M2-like TAM polarization through S1P signaling (Xie et al., 2025). In pancreatic ductal adenocarcinoma, PI3Kγ regulates myeloid-derived suppressor cell (MDSC) recruitment and suppresses anti-tumor immunity, whereas combining PI3Kγ inhibition with an ENO1 DNA vaccine augments B cell-mediated immune responses, increases CD8^+^ T cell and M1-like TAMs infiltration, and suppresses Tregs and angiogenesis, indicating PI3Kγ as a critical signaling node in TAM/MDSC-mediated immune regulation (Curcio et al., 2024). In pancreatic ductal adenocarcinoma, PI3Kγ inhibition combined with an ENO1 DNA vaccine increases CD8^+^ T cell and M1-like TAMs infiltration while reducing Tregs and angiogenesis (Kierans and Taylor, 2024). In diffuse large B-cell lymphoma, tumor-derived exosomal ENO2 activates the GSK3β/β-catenin/c-Myc axis, enhances glycolysis, and promotes M2 macrophage polarization (Shao et al., 2024). A key functional consequence of the macrophage shift toward glycolysis is increased ATP production and pentose phosphate pathway activity to support biosynthesis (O'Neill and Pearce, 2016). Citrate, a tricarboxylic acid cycle intermediate, is generated in mitochondria from oxaloacetate and acetyl-CoA by citrate synthase (Liu et al., 2017). In pro-inflammatory macrophages, glycolysis is also connected to citrate metabolism through remodeling of the tricarboxylic acid cycle. Citrate can accumulate and be exported from mitochondria to the cytosol, where it supports fatty acid synthesis, prostaglandin production, and inflammatory mediator generation (Mao et al., 2022). Therefore, citrate is not only a downstream metabolic product of glucose metabolism but also an important signaling-related metabolite that links glycolytic activation to inflammatory macrophage functions.

### 3.2 Gluconeogenesis

Gluconeogenesis produces glucose or glucose-6-phosphate from non-carbohydrate substrates, including lactate, glycerol, and glutamine (Hatting et al., 2018). Compared with glycolysis, gluconeogenesis has received less attention in TAM biology. Inflammatory M1-like TAMs can use gluconeogenesis to convert tricarboxylic acid cycle intermediates into glucose-derived metabolites, thereby maintaining intracellular glucose balance (Hatting et al., 2018). In melanoma, tumor-derived versican activates the TLR2-MyD88-RelB pathway and upregulates pyruvate carboxylase (PCB) in TAMs (Shu et al., 2025). Human macrophages and TAMs can also use glycogen metabolism and gluconeogenesis through enzymes such as phosphoenolpyruvate carboxykinase 2, mitochondrial (PCK2), and fructose-bisphosphatase 1 (FBP1). These enzymes allow macrophages to convert glutamine, lactate, and glycerol into metabolic intermediates that support cytokine production and phagocytosis (Jeroundi et al., 2024). STS has been shown to regulate the FBP1/Akt/Rab27a axis in TAMs, reducing exosomal PD-L1 secretion and promoting anti-tumor polarization. This enhances phagocytosis, activates CD8^+^ T cells, and improves the response to PD-1/PD-L1 blockade (Cheng et al., 2025). However, FBP1 does not always support anti-tumor immunity. In another context, cancer-associated fibroblast-induced MDSCs secrete FBP1-rich exosomes that suppress T cell proliferation and function. Blocking FBP1 or exosome release partially restores immune activity (Zhu et al., 2023). In TAMs, FBP1 may support anti-tumor macrophage activity, whereas in MDSC-derived exosomes it may contribute to immune suppression (Zhu et al., 2023). Overall, gluconeogenesis may help TAMs maintain pro-inflammatory activity and phagocytosis in glucose-limited TMEs (Zhu et al., 2023). Unlike glycolysis, which is often associated with rapid activation and lactate production, gluconeogenesis may support metabolic adaptation and immune maintenance. Further studies are needed to clarify when this pathway promotes anti-tumor immunity and when it contributes to immune suppression.

### 3.3 PPP

The PPP is an important branch of glucose metabolism. Its oxidative arm produces NADPH, while its non-oxidative arm generates ribose-5-phosphate for nucleotide synthesis (Sharkey, 2021). In macrophages, NADPH is required for redox balance, oxidative bursts, lipid synthesis, and inflammatory responses (Tao et al., 2017). Therefore, the PPP can support both immune activation and metabolic adaptation. G6PD is the rate-limiting enzyme of the oxidative PPP. In triple-negative breast cancer, glucose-6-phosphate dehydrogenase (G6PD) interacts with phosphorylated STAT1 to increase CCL2 and TGF-β1 expression, thereby promoting M2-like TAM polarization (Li et al., 2023). These TAMs then secrete IL-10 and activate tumor cells in a positive feedback loop, enhancing tumor proliferation and migration (Li et al., 2023). In hepatocellular carcinoma, an enzyme-based immune gene prognostic signature, including G6PD, ENO1, and SLC2A1, is mainly expressed in macrophages. Patients with high-risk scores show increased TAM infiltration, stronger immune evasion, and altered drug sensitivity (Pu et al., 2025). Another PPP enzyme, PGLS, is overexpressed in several cancers and is associated with poor prognosis. PGLS promotes M2-like TAM and Treg accumulation while reducing M1 macrophages and CD8^+^/CD4^+^ T cell infiltration, thereby creating an immunosuppressive TME (Lei et al., 2025). Compared with G6PD, PGLS has been less studied, but available data suggest that it may also participate in TAM-driven immune remodeling. Taken together, the PPP provides TAMs with reducing power and biosynthetic precursors.

### 3.4 Glycogen metabolism

Glycogen is the storage form of glucose and can be mobilized when extracellular glucose is limited (Li et al., 2024). Both M1-and M2-like TAMs can store glycogen (Roach et al., 2012); M1-like TAMs may rely on glycogen mobilization to sustain inflammatory activity and phagocytosis, whereas M2-like TAMs may use glycogen to support long-term survival, tissue repair-like functions, and immunosuppression (DiNuzzo et al., 2019). In lung adenocarcinoma, high expression of glycogen branching enzyme 1 (GBE1) is associated with poor prognosis and positively correlates with CD163^+^ TAM infiltration (Liang et al., 2021). In the TME, glycogen phosphorylase PYGL is highly expressed in TAMs and promotes lactate metabolism, suppresses M1-like polarization, and reduces CD8^+^ T cell infiltration, thereby supporting tumor immune escape (Zhao et al., 2021). Similarly, in rectal adenocarcinoma, PYGM is overexpressed in malignant cells and promotes M2-like TAM polarization, tumor proliferation, migration, and poor response to immunotherapy (Xu et al., 2025). These findings indicate that glycogen metabolism may help TAMs and tumor cells adapt to nutrient stress.

### 3.5 Pyruvate metabolism

Pyruvate is the end product of glycolysis and can be converted into lactate, enter the TCA cycle, or contribute to fatty acid synthesis (Mendz et al., 1994). In TAMs, pyruvate metabolism is closely linked to polarization. M1-like TAMs often convert pyruvate into lactate to maintain glycolysis-dependent inflammatory activity, whereas M2-like TAMs more often channel pyruvate into mitochondrial OXPHOS to support immunosuppressive and tissue repair-like functions (Busard et al., 1989). Lactate is one of the most important pyruvate-derived metabolites in the TME. Rhein-DCA enhances the inhibitory effect of DCA on PDK1, disrupts mitochondrial OXPHOS, reduces lactate levels, and reverses M2-like TAM polarization polarization of TAMs (Zhang et al., 2025). In pituitary adenomas, tumor-derived lactate induces M2-like TAM polarization through mTORC2/ERK activation and CCL17 secretion, which promotes tumor invasion through the CCL17/CCR4/mTORC1 axis (Vekariya et al., 2018). In glioblastoma, LDHA supports a tumor–macrophage metabolic loop. Tumor LDHA increases CCL2 and CCL7 through ERK/YAP1/STAT3 signaling, recruiting TAMs, while TAM-derived LDHA-containing exosomes further enhance tumor glycolysis and proliferation (Khan et al., 2024). In gliomas, high LDHA and MCT1 expression increases lactate production. Lactate then activates the receptor GPR65 on TAMs, leading to cAMP/PKA/CREB signaling, HMGB1 secretion, and a feedback loop that promotes tumor growth and stromal remodeling (Yan et al., 2024). In clear cell renal cell carcinoma, LDHA-mediated exosomal EPHA2 activates PI3K/AKT/mTOR signaling in macrophages, induces M2-like TAM polarization, and promotes tumor growth, invasion, and migration (Zhou et al., 2023). Overall, pyruvate metabolism links glycolysis, lactate signaling, mitochondrial metabolism, and macrophage polarization.

### 3.6 Hypoxia

Hypoxia is a common feature of the TME and strongly affects TAM metabolism. Under hypoxic conditions, HIF-1α activates genes involved in glucose uptake, glycolysis, lactate production, and extracellular acidification (Burtscher et al., 2025). In TAMs, HIF-1α can promote glycolysis and lactate production, favoring M2-like immunosuppressive polarization and supporting tumor growth and angiogenesis. Thus, HIF-1α links metabolic stress with immune suppression (Liu et al., 2025). In colorectal cancer, SPP1^+^ TAMs show high NAMPT expression, which stabilizes HIF-1α, promotes M2-like TAM polarization, and reinforces immunosuppression (Qu et al., 2025). In contrast, NAMPT deficiency activates STING signaling and type I interferon responses, thereby enhancing CD8^+^ T cell activity (Qu et al., 2025). In ovarian cancer, PLIN2^+^ TAMs in ascites maintain lipid metabolism and M2 like TAM polarization through the HIF-1α/SPP1 axis, promoting tumor migration, invasion, and ascites-related metastasis (Luo et al., 2025). These studies suggest that hypoxia-related pathways often cooperate with lipid and glucose metabolism to sustain immunosuppressive macrophage states.

## 4 Therapeutic targeting of glucose metabolism in TAMs

Because glucose metabolism shapes TAM polarization and immune function, it has become an attractive therapeutic target. Current strategies mainly include glycolysis inhibition, blockade of lactate metabolism, targeting of key metabolic enzymes, and combination with immunotherapy. However, these approaches must be applied carefully, because glucose metabolism is also essential for activated T cells and other anti-tumor immune cells. Glycolysis inhibitors, such as 2-deoxy-D-glucose (2-DG), can suppress glucose utilization in both tumor cells and TAMs. This may reduce pro-tumor metabolites and limit M2-like TAMs polarization (Pajak et al., 2019). However, broad inhibition of glycolysis may also impair effector T cell activity. Therefore, future strategies should aim to improve cell selectivity or combine glycolytic inhibition with immune-supportive approaches. Targeting lactate metabolism may offer a more focused way to reduce TAM-mediated immunosuppression. Inhibition of LDHA or PDK can lower lactate production, reduce lactate-driven M2-like TAMs polarization, and relieve immunosuppressive signaling (Zhang T. et al., 2019). Compared with general glycolysis inhibition, lactate pathway blockade may better target the immunosuppressive metabolite network of the TME.

Glutamine metabolism also interacts with TAM polarization (Wen et al., 2025). In urological cancers, TAM glutamine metabolism maintains an M2-like phenotype through TNF/mTORC1 signaling (Karonitsch et al., 2018). The glutamine antagonist JHU083 blocks TAM glutamine utilization, promotes M1-like reprogramming, enhances tumor cell phagocytosis, suppresses angiogenesis, and also interferes with tumor cell HIF-1α/c-MYC signaling (Karonitsch et al., 2018). This dual effect suggests that metabolic inhibitors may work best when they affect both tumor cells and immunosuppressive myeloid cells. Combining metabolic intervention with immunotherapy is particularly promising. Metabolic modulation can reprogram TAMs from an M2-like state toward an M1-like phenotype, restore phagocytosis, and enhance CD8^+^ T cell and NK cell activation (Kyrysyuk and Wucherpfennig, 2023). Several studies show that targeting glycolysis or lactate pathways reduces TAM-mediated immunosuppression, improves anti-PD-1/PD-L1 responses, slows tumor growth, and inhibits metastasis (Guo et al., 2022).

## 5 Conclusion

Glucose metabolism is a key regulator of TAM functional plasticity, influencing not only energy production and biosynthesis but also macrophage polarization, cytokine secretion, phagocytosis, antigen presentation, and T cell interactions (Yang et al., 2024). Rather than acting as a simple M1-/M2-like switch, TAM glucose metabolism reflects a dynamic response to tumor-derived signals, hypoxia, nutrient stress, stromal interactions, and therapeutic pressure (Zhou et al., 2022). Pathways including glycolysis, lactate metabolism, the PPP, gluconeogenesis, glycogen metabolism, and pyruvate metabolism may exert context-dependent effects, either supporting pro-inflammatory activity or promoting immunosuppressive TAM programs within the TME. Therefore, precise metabolic reprogramming of TAMs may offer a promising strategy to restore anti-tumor immunity and improve responses to immunotherapy, particularly in combination with immune checkpoint blockade.
